# Merit of Anisodamine Combined with Opioid *δ*-Receptor Activation in the Protection against Myocardial Injury during Cardiopulmonary Bypass

**DOI:** 10.1155/2013/212801

**Published:** 2013-12-10

**Authors:** Xuan Hong, Huimin Fan, Rong Lu, Paul Chan, Zhongmin Liu

**Affiliations:** ^1^Department of Cardiothoracic Surgery, Shanghai East Hospital, Tongji University, 150 Jimo Road, Shanghai 200120, China; ^2^Division of Cardiovascular Medicine, Department of Internal Medicine, Wan Fang Hospital, Taipei Medical University, 111 Hsin-Lung Road, Section 3, Taipei 116, Taiwan

## Abstract

Myocardial ischemia/reperfusion (MIR) injury easily occurrs during cardiopulmonary bypass surgery in elderly patients. In an attempt to develop an effective strategy, we employed a pig model of MIR injury to investigate the maximum rate of change of left ventricular pressure, left ventricular enddiastolic pressure, and left intraventricular pressure. Coronary sinus cardiac troponin T (TnT) and adenosine-triphosphate (ATP) content in myocardium were measured. The ultrastructures for MIR injury were visualized by transmission electron microscopy (TEM). The role of *δ*-opioid receptor activation using D-Ala2, D-Leu5-enkephalin (DADLE) in both early (D1) and late (D2) phases of cardioprotection was identified. Also, the merit of cardioprotection by DADLE in combination with anisodamine, the muscarinic receptor antagonist (D+M), was evaluated. Glibenclamide was employed at the dose sufficient to block ATP-sensitive potassium channels. Significant higher cardiac indicators, reduced TnT and increased ATP contents, were observed in D1, D2, and D+M groups compared with the control group. DADLE induced protection was better in later phase of ischemia that was attenuated by glibenclamide. DADLE after the ischemia showed no benefit, but combined treatment with anisodamine showed a marked postischemic cardioprotection. Thus, anisodamine is helpful in combination with DADLE for postischemic cardioprotection.

## 1. Introduction 

Myocardial ischemia/reperfusion (MIR) injury is a major determinant of therapeutic outcome during cardiac surgery with cardiopulmonary bypass or before and after cardiac interventional therapy. Many studies have investigated the pathogenesis of MIR. However, MIR injury remains a high-risk factor which affects the therapeutic efficacy of surgical procedures in elderly patients with severe cardiac disease. So it is still significant to develop the effective treatment for MIR.

It is now recognized that ischemic preconditioning (IPC) mitigates MIR injury [1]. Endogenous mediators including opiates, adenosine, and bradykinin are considered to promote the acute IPC which can protect not only against myocardial stunning but also ischemia-induced myocardial injury. Cardioprotection provided by IPC has been divided into early (the first protective window) and late phases (the second protective window) as described previously [2]. The early-phase of protection develops within minutes of the initial IPC and lasts 1 to 2 hours, while the late phase becomes apparent 24 h later and lasts 3 to 4 days. Because of its sustained duration (30–90 min) and the limitation of traditional surgical IPC in clinical application (i.e., clamping the aorta many times before blocking), the protective effect of preconditioning induced by opioid *δ*-receptor agonist(s) has been indicated [3].

There are three well-characterized families of opioid peptides produced by the body: enkephalins, dynorphins, and *β*-endorphins, which act at corresponding *δ*, *κ*, and *μ* receptors. These belong to a group of Gi/Go protein-coupled receptors. Two types of *δ*-opioid receptor (*δ*_1_ and *δ*_2_), three types of *κ* receptors (*κ*_1_, *κ*_2_, and *κ*_3_), and two *μ* receptors (*μ*_1_ and *μ*_2_) have been identified. The expression of *δ* and *κ* receptors has been reported in the heart [3].

Previous data show that preconditioning induced by opioid receptor agonists such as morphine, TAN-67 and D-Ala2, D-Leu5-enkephalin (DADLE) before acute myocardial infarction may stimulate the effect of IPC on heart in mouse, dog, rabbit, and pig models [4–9] and not only promotes the recovery of heart function after acute myocardial infarction, but also initiates myocardial protection effect 24 h after preconditioning. Further studies indicate that the most notable merit of *δ*-opioid receptor activation is to provide cardioprotection [10–14].

The increase in acetylcholine (ACh) during myocardial reperfusion has been demonstrated to be one of the leading causes of myocardial injury. This has led to the postulation of the ACh-Ca^2+^-OFR axis theory [15, 16]. Administration of scopolamine results in the blockade of muscarinic receptors. In this way it inhibits the ACh-Ca^2+^-OFR axis, which protects the energy metabolism of myocardial cells and the integrity of myocardial ultrastructure, which in turn protects the myocardium [15, 16]. As a potential treatment strategy, administration of *δ*-opioid receptor agonist alone or in combination with muscarinic antagonist may play an important role in the cardioprotection.

The present study employed a pig model with MIR injury during cardiopulmonary bypass to investigate the myocardial protective effects of drug therapy. Using combined therapy with anisodamine, a naturally occurring atropine-like compound that has been characterized in China [17–19], we explored the early and late phases of preconditioning with DADLE, the opioid *δ*-receptor agonist, in an attempt to provide theoretical and experimental evidence for further clinical application in cardioprotection.

## 2. Material and Methods

### 2.1. General Surgical Preparation

Healthy pigs of the Shanghai strain, each weighing 30–35 kg, were purchased from the Shanghai Baomu Laboratory Animal Center. Following basal anesthesia (peritoneal injection with 30 mg/kg pentobarbital and intramuscular injection 0.3–0.5 mg/kg diazepam), venous access was established via the auricular vein. General anesthesia was maintained by intravenous injection with 1 mg/kg Diprivan and intramuscular injection of 0.6 mg/kg tracrium. The femoral vein cannula was connected to a ALC-MPA multichannel biological signaling analysis system to monitor mean arterial pressure. The trachea was incised and intubated (tube with internal diameter of 75–78 mm). Ventilation was controlled as follows: oxygen : air 1 : 1; tidal volume, 10 mg/kg; respiratory rate, 13 times/min; oxygen concentration, 50%; airway pressure, 15–20 cm H_2_O. Blood gas analysis was performed regularly, and the stability of the internal environment was sustained.

A median sternotomy was performed, and the heart was exposed. A tube was implanted into the apex and then connected with the ALC-MPA (Shanghai Alcott Biotech Co., Ltd., China) multichannel signaling system to measure the left ventricular systolic pressure (LVSP), left ventricular enddiastolic pressure (LVEDP), and the maximum rate of change of left ventricular pressure (±dp/±dtmax).

Heparin (3 mg/kg) was given, and the ascending aorta, superior and inferior vena cava, and coronary sinus were intubated. An artificial heart-lung machine (precharged with crystal) with bubble oxygenators was used to establish the cardiopulmonary bypass. When the aorta was blocked, a modified St. Thomas' solution (the buffer consisted of the following in millimoles per liter: NaCl, 118; KCl, 4.7; MgSO_4_, 1.2; KH_2_PO_4_, 1.2; NaHCO_3_, 25; CaCl_2_, 2.5; Na_2_EDTA, 0.5; and glucose, 11; pH 7.4.) at a concentration of 5 mL/kg was perfused into myocardium. An ice bath was applied around the heart to drop temperature and arrest the heart. During the period of cardiopulmonary bypass, the temperature of nose and pharynx was sustained at 28°C, aortic perfusion pressure at 6.67 kPa, and arterial oxygen partial pressure at 20–30 kPa. After 60 min of cardioplegic ischemia, the aorta was opened, and the ischemic myocardium was reperfused. The temperature was increased, and electric defibrillation was employed with a power of 20 J. Cardiotonic agents including dopamine and adrenaline were given to attain hemodynamic stability, after which the cardiopulmonary bypass was withdrawn. The pigs were sacrificed 2 h after the termination of cardiopulmonary bypass.

This study was approved by the Ethics Review Committee of Shanghai East Hospital, Tongji University, and all animal handling procedures were performed according to the Guide for the Care and Use of Laboratory Animals of the National Institutes of Health, as well as the guidelines of the Animal Welfare Act.

### 2.2. Animal Groups and Treatment Protocols

Pigs were randomly assigned to five groups: C, D1, D2, D+K, and D+M. Group C was the control for the model of cardiopulmonary bypass. Pigs in Group D1 were intravenously injected with 1 mg/kg DADLE 1 h before cardiopulmonary bypass. Animals in Group D2 were intravenously injected with 1 mg/kg DADLE 48 and 24 h before cardiopulmonary bypass. Pigs in Group D+K were intravenously injected with 1 mg/kg DADLE combined with 1 mg/kg glibenclamide 1 h before cardiopulmonary bypass. Animals in Group D+M were intravenously injected with 1 mg/kg DADLE combined with 0.5 mg/kg anisodamine 1 h before cardiopulmonary bypass, followed by addition of 0.5 mg/kg anisodamine. The left ventricular systolic pressure (LVSP), left ventricular end diastolic pressure (LVEDP), and ±dp/±dtmax were measured before and 1 and 2 h after termination of cardiopulmonary bypass.

Coronary sinus blood was collected before and after cardiopulmonary bypass, at aortic opening and 1 and 2 h after termination of cardiopulmonary bypass. The plasma was used for detection of cardiac troponin T (TnT). The left ventricular myocardium was sampled before cardiopulmonary bypass and 2 h after termination of cardiopulmonary bypass. Some of the samples were stored in liquid nitrogen for the subsequent determination of the adenosine-triphosphate (ATP) content in myocardial tissues. Others were stored in glutaraldehyde solution at 4°C for observation of ultrastructural changes using transmission electron microscopy.

### 2.3. Determination of TnT Values in Coronary Sinus Blood

TnT values in coronary sinus blood were detected using the electrochemical luminescence method on an Elecsys 1010 Chemistry Analyzer (Roche, Basel, Switzerland).

### 2.4. Detection of Gi*α* Protein Expression and PKC Activity in Myocardial Tissues

The expression of Gi*α* protein and PKC activity in myocardial tissues were detected using western blotting analysis. Briefly, myocardial tissues were lysed, homogenized, and centrifuged and the supernatant collected. Protein concentration was measured according to a standard curve created using bovine serum albumin. This was followed by sodium dodecyl sulfate polyacrylamide gel electrophoresis (SDS-PAGE) and immunoblotting. The membrane was then transferred to Ponceau S staining solution to observe the protein transfer. Finally, the membrane was treated with specific antibodies (mouse anti-human PKC or Gi*α* protein) and visualized using a Storm 840 Gel and Blot Imaging System.

### 2.5. Determination of ATP Content in Myocardial Tissues

ATP content was determined using the high-performance liquid chromatography (HPLC) on LC-10A Semi-Micro Liquid Chromatographic System (Shimadzu, Kyoto, Japan).

### 2.6. Changes of Morphology and Ultrastructure of Myocardial Tissues

At the end of the experiment, a section of left ventricular myocardium was sampled and immediately fixed in glutaraldehyde solution at 4°C. Sections were prepared following routine procedures, and the changes in morphology and ultrastructure of the myocardial tissues were observed under a transmission electron microscopy.

### 2.7. Statistical Analysis

All data were expressed as the mean ± standard deviation (SD) of each group. Analyses were performed using Statistical Analysis System (SAS8.0) software. Analysis of variance was used to compare differences between treatment groups. *P*-values < 0.05 were considered statistically significant.

## 3. Results

### 3.1. Index of Heart Function

Changes in cardiac function parameters are summarized in Table 1. Statistical significance of higher LVSP values was observed in groups D1 and D2, compared with that in Group C after cardiopulmonary bypass, 1 h after termination of cardiopulmonary bypass (*P* < 0.05), and 2 h after termination of cardiopulmonary bypass (*P* < 0.01). LVEDP values were significantly higher in groups D1, D2, and D+M than in groups C or D+K at 1 h after termination of cardiopulmonary bypass (*P* < 0.05) and values in groups D1, D2, and D+M were significantly higher than those in Group C at 2 h after termination of cardiopulmonary bypass (*P* < 0.01). However, 2 h after the termination of cardiopulmonary bypass, LVEDP was lower in Group D2 than in Group D1 (*P* < 0.05).

The maximum rate of rise of left ventricular pressure (+dp/+dtmax) was significantly higher in Group D1 than that in Group C after cardiopulmonary bypass and 1 h after termination of cardiopulmonary bypass (*P* < 0.05) or 2 h after termination of cardiopulmonary bypass (*P* < 0.01). Values of left ventricular pressure (+dp/+dtmax) were markedly higher in Group D2 than in Group C after cardiopulmonary bypass (*P* < 0.05) and at 1 or 2 h after the termination of cardiopulmonary bypass (*P* < 0.01). In addition, +dp/+dtmax values were significantly higher in Group D+M than in Group C after cardiopulmonary bypass, and 1 or 2 h after the termination of cardiopulmonary bypass (*P* < 0.05).

At 1 and 2 h after termination of cardiopulmonary bypass, the absolute values of the maximum rate of fall of left ventricular pressure (−dp/−dtmax) were significantly lower in Group C than those in Group D1 (*P* < 0.05). In addition, lower absolute values were observed in Group C than in Groups D2 and D+M after cardiopulmonary bypass, and 1 or 2 h after the termination of cardiopulmonary bypass (*P* < 0.01). At 2 h after termination of cardiopulmonary bypass, the absolute value of −dp/−dtmax was significantly higher in Group D2 as compared to Group D1 (*P* < 0.05).

### 3.2. TnT Values in Coronary Sinus Blood

As shown in Table 2, the marked lower TnT values were observed in Groups D1, D2, and D+M in comparison with those in Group C at the time of aortic opening, after cardiopulmonary bypass and 1 or 2 h after the termination of cardiopulmonary bypass (*P* < 0.01). In addition, TnT values were more significantly reduced in Group D2 than in Group D1 at 1 or 2 h after the termination of cardiopulmonary bypass (*P* < 0.01).

### 3.3. ATP Content in Myocardial Tissues

The ATP content in myocardial tissue was significantly higher in Groups D1, D2, and D+M, compared with that in Group C (*P* < 0.01). However, ATP content was lower in Group D1 as compared to Groups D2, D+M, or normal myocardium (*P* < 0.05; Table 3).

### 3.4. Gi*α* Protein Expression and PKC Activity in Myocardial Tissues

**Figure 1:**
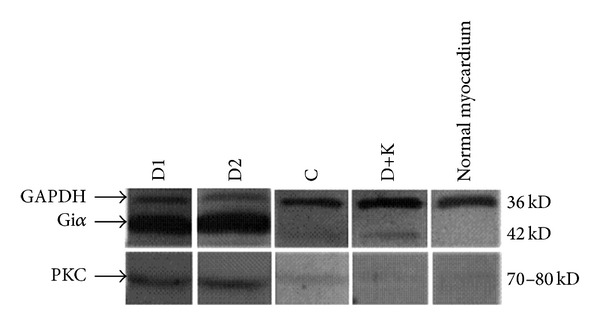
Expression of Gi*α* protein in myocardial tissues and PKC activity in myocardial tissues (1), Group D1; (2) Group D2; (3) Group C; (4) Group D+K; (5) normal myocardium.

Higher expression of Gi*α* or PKC protein in myocardial tissue was observed in Groups D1 and D2 than in Group C (*P* < 0.01). Representative picture was shown in Figure 1 and the data summarized in Table 4.

### 3.5. Changes in Morphology and Ultrastructure of Myocardial Cells

**Figure 2:**
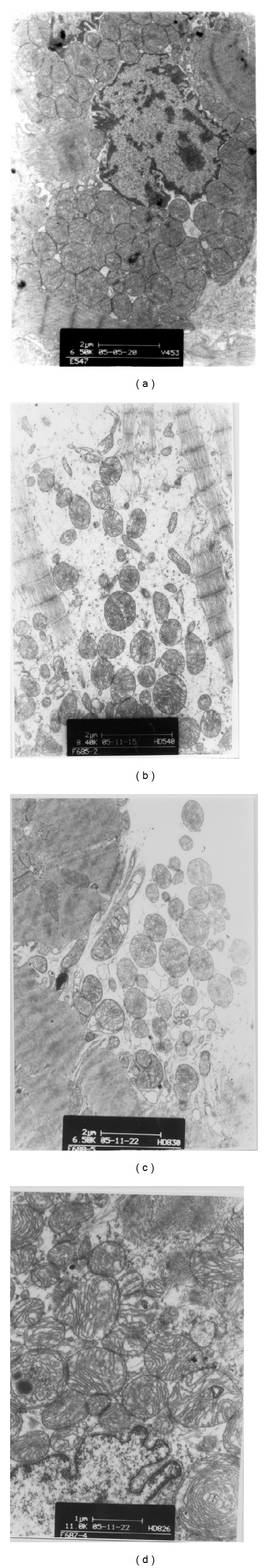
The visual appearance of myocardial cells of pigs. (a) In Group C (×6500). (b) In Group C (×8400). (c) In Group D+K (×6500). (d) In Group D+K (×11000).

**Figure 3:**
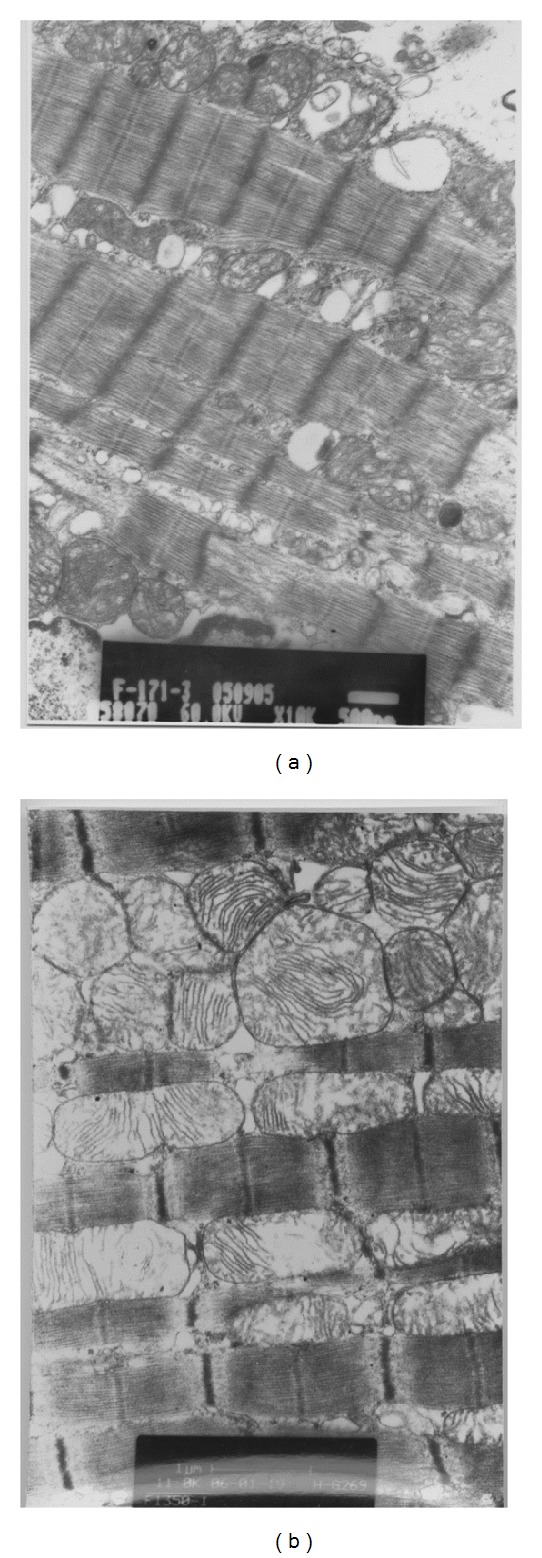
The ultrastructure of myocardial cells of pigs. (a) In Group D1 (×10000). (b) In Group D1 (×11000).

**Figure 4:**
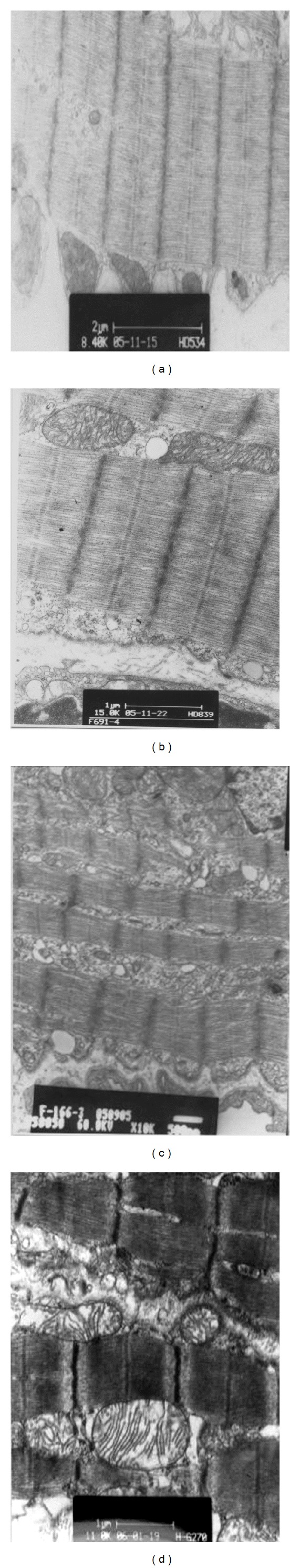
The ultrastructure of myocardial cells of pigs. (a) In Group D2 (×8400). (b) In Group D2 (×15000). (c) In Group D+M (×10000). (d) In Group D+M (×11000).

Transmission electron microscopy revealed the rupture of muscular fibers, together with mitochondrial swelling, and intracellular edema in Groups C and D+K. In addition, the shape of nucleus was irregular, with evidence of mitochondrial overflow after cell death (Figure 2). By contrast, in Group D1, few muscular fibers were ruptured, with only mild swelling of mitochondria, mild intercellular edema, and no cell death (Figure 3). In Groups D2 and D+M, the ruptured muscular fibers, mitochondrial or intracellular edema, and dead cells were all not observed (Figure 4).

## 4. Discussion

For the role of opioid receptors in cardioprotection, preconditioning with *δ*-opioid receptor agonists such as DADLE has been shown to produce merit in mouse, dog, rabbit, and pig models [4–9]. Also, *κ*-opioid receptor agonists exerted a direct cardioprotective effect against ischemia/reperfusion [12]. Moreover, *δ*_2_-opioid receptor manipulation interferes with the ability of deltorphin E (a *δ*_2_-opioid receptor agonist) to increase survival after hemorrhage [13]. Preconditioning with morphine administered into the spinal canal of rats indicated that *μ*, *δ*, and *κ* receptors play important roles in myocardial protection [9, 14]. However, peripheral *δ*_2_-opioid receptor activation induced cardiac tolerance to ischemia/reperfusion injury *in vivo*, while agonists of *μ*, *δ*_1_, *κ*_1_, and *κ*_2_ receptors did not [20]. This finding forms the basis for additional investigation into the mechanisms by which opioid receptors facilitate cardioprotection. The most robust cardioprotection is introduced to be mediated by *δ* receptors, particularly *δ*_2_-opioid receptors [9, 10]. However, there are no studies reporting the role of DADLE, a *δ*-opioid receptor agonist, in cardiopulmonary bypass models during cardiac surgery.

The present study established a pig model of myocardial ischemia/reperfusion injury with cardiopulmonary bypass to investigate the cardioprotection of *δ*-opioid receptors. We also explored the protection in early and late phases of preconditioning with DADLE to provide the experimental evidences for novel treatment strategies of myocardial protection.

In the present study, the protective effects of DADLE against myocardial ischemia/reperfusion injury during cardiopulmonary bypass were observed because preconditioning with DADLE significantly reduced the release of TnT, preserved ATP within myocardial cells, increased the systolic and diastolic functions of myocardium, and promoted the recovery of myocardial function after myocardial ischemia/reperfusion injury. We found that administration of DADLE 48 and 24 h before cardiopulmonary bypass achieved a significant improvement in each of the indicators compared with the control group. It also induced late-phase cardioprotection with better protection of the myocardial ultrastructure and improved the diastolic function of heart. Such findings provide the experimental basis for developing a control strategy for myocardial protection in clinical practices.

It has recently been documented that use of nitric oxide synthase (NOS), PKC, and K_ATP_ inhibitors all antagonized *δ*_2_-opioid receptor-mediated protection against myocardial ischemia/reperfusion injury [21]. The present study showed that preconditioning with DADLE induced a higher expression of Gi*α* or PKC protein. This is consistent with the previous report [22, 23]. We also demonstrated that blockade of K_ATP_ channel abolished the actions of DADLE and no significant differences were observed in the indicators of heart function such as TnT values, ATP content, and transmission electron microscopical findings, compared with the control group. We, therefore, speculate that Gi*α* protein, PKC, and K_ATP_ channels play important roles in *δ*-opioid receptor-mediated cardioprotection against myocardial ischemia/reperfusion injury during cardiopulmonary bypass.

Our results are consistent with the previous reports, such as the role of Gi/Go proteins in *δ*-opioid receptor-mediated cardioprotection [3]. Also, the early-phase cardioprotection of DADLE was abolished by two PKC inhibitors, chelerythrine and GF109203X [24]. Also, the activation of opioid receptors elicited late-phase cardioprotection in rat ventricular myocytes and was inhibited by chelerythrine, suggesting that opioid receptor-elicited late-phase cardioprotection was induced in a PKC-dependent manner [25]. It has been documented that PKC-*β*_1_ translocated to the nucleus for late-phase signaling transduction, on the basis that transcription and translation of the late-phase were dependent on nuclear factors [24]. Thus, DADLE may induce the translocation of PKC isoform such as PKC-*α* to the sarcolemma, PKC-*δ* to the mitochondria, and PKC-*ε* to the intercalated disk and mitochondria. Then, K_ATP_ channel can be regulated by PKC as described in previous report [26, 27]. This is the main signal pathway for DADLE induced cardioprotection.

Moreover, the present study showed the merit in combination with anisodamine for protective effect of preconditioning with DADLE against myocardial ischemia/reperfusion injury during cardiopulmonary bypass.

It has been identified that the increased ACh during myocardial ischemia/reperfusion was one of the major causes of myocardial injury to result in the ACh-Ca^2+^-OFR axis theory [15, 16]. By inhibiting M receptors, scopolamine causes ACh to accumulate in postsynaptic gaps which in turn feeds back to presynaptic M receptors and inhibits the further release of ACh. In this way, scopolamine not only blocks the actions of released ACh, but also reduces its further release. This dual action inhibits the ACh-Ca^2+^-OFR axis protects the energy metabolism of myocardial cells and the integrity of myocardial ultrastructure, which in turn protects the myocardium [15, 16]. Anisodamine is similar to scopolamine to block M receptors [28]. Anisodamine is similar to scopolamine to block M receptors [29, 30] with a chemical structure of 7*β*-hydroxyhyoscyamine [28]. Also, anisodamine is used to treat endotoxic shock [31–33]. It is, therefore, considered that combined administration of DADLE and anisodamine prior to the surgery is a feasible approach to prevent the occurrence of myocardial ischemia/reperfusion injury. Actually, we found that combined treatment with DADLE and anisodamine exerts a powerful cardioprotective effect and this view has not been mentioned before.

## 5. Conclusion 

In summary, administration of DADLE 48 and 24 h before cardiopulmonary bypass elicited significantly higher late-phase cardioprotection and promoted the recovery of heart function after myocardial ischemia/reperfusion injury. It promoted the recovery of heart function after myocardial ischemia/reperfusion injury, decreased the release of TnT, preserved ATP within myocardial cells, and protected the integrity of myocardial ultrastructure. Combined treatment with DADLE and anisodamine results in a more powerful cardioprotection than DADLE only. Blockade of K_ATP_ channels with glibenclamide significantly inhibited this *δ*-opioid receptor-mediated early-phase cardioprotection in pig models, indicating that the Gi*α*- PKC- K_ATP_ channel pathway is important in *δ*-opioid receptor-mediated cardioprotection after myocardial ischemia/reperfusion injury during cardiopulmonary bypass in pigs.

## Figures and Tables

**Table 1:** Changes of indicators of heart functions.

Group	LVSP (kPa)				LVEDP (kPa)				Maximum rate of rise of left ventricular pressure (kPa/s)				Maximum rate of fall of left ventricular pressure (kPa/s)			
	Before CPB	After CPB	1 h after termination of CPB	2 h after termination of CPB	Before CPB	After CPB	1 h after termination of CPB	2 h after termination of CPB	Before CPB	After CPB	1 h after termination of CPB	2 h after termination of CPB	Before CPB	After CPB	1 h after termination of CPB	2 h after termination of CPB
C	13.04 ± 3.19	11.23 ± 4.64	12.82 ± 3.84	12.13 ± 2.60	2.06 ± 0.90	2.10 ± 0.51	3.75 ± 1.78**	5.02 ± 1.42**	328.33 ± 67.67	343.54 ± 142.42	354.03 ± 142.42	339.71 ± 161.71	−204.73 ± 45.43	−103.13 ± 48.33**	−86.15 ± 42.95**	−79.41 ± 45.22**
D1	13.53 ± 1.75	16.12 ± 1.43^∗#^	17.22 ± 2.89^∗#^	16.42 ± 2.52^∗#^	2.10 ± 0.52	2.07 ± 0.39	2.01 ± 0.42^#^	2.67 ± 0.60^∗##^	322.46 ± 63.08	475.57 ± 78.19^∗∗#^	540.11 ± 90.56^∗∗#^	584.36 ± 106.78^∗∗##^	−224.27 ± 66.57	−155.12 ± 50.12*	−146.66 ± 39.75^∗#^	−135.10 ± 34.31^∗#^
D2	13.73 ± 2.74	16.98 ± 3.0^∗#^	16.15 ± 2.06^∗#^	16.87 ± 3.07^∗#^	1.68 ± 0.51	1.66 ± 0.58	1.94 ± 0.21^#^	2.10 ± 0.23^∗##△^	361.79 ± 150.29	606.74 ± 247.68^∗∗#^	613.37 ± 107.61^∗##^	690.20 ± 245.27^∗∗##^	−237.43 ± 59.79	−175.66 ± 55.26^#^	−182.91 ± 41.07^##^	−192.34 ± 45.19^##△^
D+K	13.34 ± 3.22	14.64 ± 4.49	14.58 ± 3.21	12.34 ± 2.60	2.03 ± 0.91	2.15 ± 0.39	3.14 ± 0.91**	3.64 ± 1.23**	309.61 ± 74.01	463.95 ± 78.33*	405.68 ± 139.28	291.85 ± 121.95	−187.26 ± 53.22	−126.55 ± 72.77**	−122.90 ± 70.88**	−121.25 ± 80.77**
D+M	14.81 ± 5.16	13.17 ± 3.13	15.35 ± 4.66	13.92 ± 4.58	1.81 ± 0.97	1.90 ± 0.93	1.97 ± 0.81^#^	2.41 ± 0.72^∗##^	328.70 ± 88.68	520.61 ± 145.64^∗∗#^	599.66 ± 153.12^∗∗#^	543.40 ± 114.86^∗∗#^	231.89 ± 37.60	178.99 ± 42.89^##^	180.00 ± 49.87^##^	165.03 ± 47.10^##^

Values shown are mean ± SD.

CPB: cardiopulmonary bypass. Compared with that before cardiopulmonary bypass, *P < 0.05, **P < 0.01. Compared with that in Group C at the same time point, ^#^ *P* < 0.05, ^##^ *P* < 0.01. ^△^ *P* < 0.01, Group D1 versus D2 at the same time point.

**Table 2:** Changes of the coronary sinus cardiac troponin T (TnT) values (ng/mL).

Group	Before CPB	Aortic opening	After CPB	After termination of CPB	
				1 h	2 h
C	0.01 ± 0.003	0.05 ± 0.03**	0.09 ± 0.04**	0.22 ± 0.10**	0.31 ± 0.08**
D1	0.01 ± 0.001	0.01 ± 0.001^#^	0.01 ± 0.01^#^	0.08 ± 0.01^∗∗#^	0.08 ± 0.02^∗∗#^
D2	0.01 ± 0.001	0.01 ± 0.001^#^	0.01 ± 0.001^#^	0.01 ± 0.001^#△^	0.01 ± 0.002^#△^
D+K	0.03 ± 0.03	0.05 ± 0.03*	0.07 ± 0.04**	0.07 ± 0.04**	0.15 ± 0.05**
D+M	0.02 ± 0.01	0.02 ± 0.01	0.04 ± 0.02^#^	0.08 ± 0.07^∗#^	0.01 ± 0.07^∗∗#^

Values shown are means ± SD.

CPB: cardiopulmonary bypass. Compared with that before cardiopulmonary bypass, *P < 0.05, **P < 0.01. Compared with that in Group C at the same time point, ^#^ *P* < 0.01. ^△^ *P* < 0.01, Group D1 versus D2 at the same time point.

**Table 3:** Changes of ATP content in myocardial tissues (*µ*mol/g tissue).

Group	ATP
C	0.90 ± 0.20
D1	1.57 ± 0.57^∗#^
D2	2.20 ± 0.46*
D+K	1.05 ± 0.17
D+M	1.67 ± 0.48*
Normal myocardium	2.25 ± 0.34*

Values shown are mean ± SD.

Compared with Group C, *P < 0.01; ^#^ *P* < 0.05, Group D1 versus Group D2 or normal myocardium.

**Table 4:** Expression of Gi*α* protein and PKC activity in myocardial tissues.

Group	Gi*α* protein	PKC
C	0.09 ± 0.02	0.39 ± 0.07
D1	0.40 ± 0.08*	0.97 ± 0.29*
D2	0.31 ± 0.08*	0.76 ± 0.09*
D+K	0.10 ± 0.04	0.30 ± 0.11
Normal myocardium	0.12 ± 0.07	0.36 ± 0.20

Values shown are mean ± SD.

Compared with Group C, *P < 0.01.
